# Morpho-phylogenetic identification and characterization of new causal agents of *Fusarium* species for postharvest fruit rot disease of muskmelon in northern Thailand and their sensitivity to fungicides

**DOI:** 10.3389/fpls.2024.1459759

**Published:** 2024-10-10

**Authors:** Nakarin Suwannarach, Surapong Khuna, Tanapol Thitla, Chanokned Senwanna, Wipornpan Nuangmek, Jaturong Kumla, Saisamorn Lumyong

**Affiliations:** ^1^ Office of Research Administration, Chiang Mai University, Chiang Mai, Thailand; ^2^ Center of Excellence in Microbial Diversity and Sustainable Utilization, Chiang Mai University, Chiang Mai, Thailand; ^3^ Department of Biology, Faculty of Science, Chiang Mai University, Chiang Mai, Thailand; ^4^ Faculty of Agriculture and Natural Resources, University of Phayao, Phayao, Thailand; ^5^ Academy of Science, The Royal Society of Thailand, Bangkok, Thailand

**Keywords:** fruit rot, fungal disease, muskmelon, pathogen identification, postharvest diseases

## Abstract

A significant global problem affecting muskmelon (*Cucumis melo* L.) is fruit rot caused by phytopathogenic fungi, which results in unsaleable products and substantial financial losses. In 2022 and 2023, fruit rot on muskmelon was found during the postharvest storage period in Phayao Province of northern Thailand. The aim of the current study was to isolate the species of fungi causing the fruit rot lesions. Out of the rot lesions on muskmelons, nine fungal isolates were received. All isolates of fungi were identified through a combination of morphological characteristics and molecular analyses. Based on their morphological traits, all isolated fungal isolate was assigned to the genus *Fusarium*. All the fungal isolates were determined to belong to the *Fusarium incarnatum-equiseti* species complex through multi-gene phylogenetic analysis employing the calmodulin (*cam*), RNA polymerase second largest subunit (*rpb2*), and translation elongation factor 1-alpha (*tef1-α*) genes. These isolates were identified as *F. compactum* (SDBR-CMU483), *F. jinanense* (SDBR-CMU484, SDBR-CMU485, and SDBR-CMU486), *F. mianyangense* (SDBR-CMU487 and SDBR-CMU488), and *F. sulawesiense* (SDBR-CMU489, SDBR-CMU490, and SDBR-CMU491). Moreover, pathogenicity tests were subsequently carried out, and the results indicated that all fungal isolates caused symptoms of fruit rot on inoculated muskmelon fruits. Notably, this result was consistent with the symptoms observed throughout the postharvest storage period. In the fungicide screening test, all fungal isolates showed sensitivity to copper oxychloride. However, all isolates showed insensitivity to benalaxyl-M + mancozeb, carbendazim, mancozeb, and metalaxy. To the best of our knowledge, the present study is the first to identify *F. compactum*, *F. jinanense*, and *F. mianyangense* as new causative agents of muskmelon fruit rot in Thailand and other regions globally. This is also the first report of postharvest fruit rot on muskmelons caused by *F. sulawesiense* in Thailand. Furthermore, the fungicide screening results indicate that fungicide resistance can be beneficial in developing potential management strategies against postharvest fruit rot disease of muskmelon caused by these four pathogenic *Fusarium* species.

## 1 Introduction

Muskmelon (*Cucumis melo* L.) is a commercially significant horticultural plant within the family Cucurbitaceae (Farcuh et al., 2020). This crop is grown globally across temperate, tropical, and subtropical areas (Wang et al., 2022a). Numerous scientific investigations have documented that muskmelon fruits serve as a nutritious source for humans. They comprise a range of essential nutritional elements such as ascorbic acid, *β*-carotene, folic acid, microelements, phenolic compounds, protein, sugars, vitamins, and several bioactive compounds (Lester and Hodges, 2008; Vella et al., 2019; Manchali et al., 2021). Additionally, they exhibit beneficial medicinal characteristics, including analgesic, anticancer, antidiabetic, antioxidant, anti-inflammatory, antimicrobial, antiulcer, diuretic, and hepatoprotective properties (Parle and Singh, 2011; Vella et al., 2019). In 2022, global melons (including cantaloupe, honeydew, and muskmelon) production reached 28.5 million tons, valued at 142.1 billion USD. China was the largest producer, contributing 14.2 million tons, followed by Turkey with 1.5 million tons, India with 1.4 million tons, and Kazakhstan with 1.2 million tons (FAOSTAT, 2022). Indonesia, being the top muskmelon producer in Southeast Asia, is followed by the Philippines and the Lao People’s Democratic Republic (FAOSTAT, 2022). Throughout the growth season, harvesting process, and post-harvest storage period, melons are susceptible to various diseases caused by bacteria, fungi, and viruses (Kwon et al., 2009; Pornsuriya and Chitphithak, 2018; Mirtalebi et al., 2019; Lima et al., 2021; de Almeida Nogueira et al., 2023; Namisy et al., 2023). Diseases can reduce both the production and quality of melon fruits, leading to customer dissatisfaction and resulting in economic losses. For example, In North-Central Mexico, diseases caused 13% of the cantaloupe melon losses in 2022 (Espinoza-Arellano et al., 2023). The average crop loss percentage for melons in 2021–2022 was 20.19% per farm in Australia. This includes 5.42% of losses occurring during and after harvest, with insufficient disease control being one contributing factor (Akbar et al., 2024). In 2022, melon losses in Iran, which range from 5% to 9% of total production, could result in economic losses of approximately 100,000 to 200,000 USD (Parsafar et al., 2023). Therefore, accurate disease identification and efficient disease control strategies could help reduce melon crop loss.

In Thailand, the area used for muskmelon production is growing since it has become one of the most significant crops for the economy (Nuangmek et al., 2019). The primary areas for cultivating muskmelons in the northern area of Thailand include Chiang Rai, Chiang Mai, Phayao, Nakhon Sawan, Phitsanulok, Phichit, and Sukhothai Provinces. Muskmelons are grown and harvested throughout the year in Thailand (Nuangmek et al., 2021; Khuna et al., 2022). Fruit rot presents a harmful disease affecting muskmelon fruits both pre-harvest and post-harvest, leading to considerable decreases in productivity and quality (Li et al., 2019; Wonglom and Sunpapao, 2020; Lima et al., 2021). Previous investigation indicates that fungi corresponding to the genera *Alternaria* (Kobayashi et al., 2004), *Diaporthe* (Broge et al., 2020), *Fusarium* (Lima et al., 2021; Khuna et al., 2022), *Lasiodiplodia* (Suwannarach et al., 2019), *Neoscytalidium* (Mirtalebi et al., 2019), *Paramyrothecium* (Huo et al., 2023), *Penicillium* (Pornsuriya and Chitphithak, 2018), *Sclerotium* (Kwon et al., 2009), and *Stagonosporopsis* (Das et al., 2023) have been associated with fruit rot in muskmelons. Additionally, the genus *Pythium*, a fungus-like microorganism, has been found to cause fruit rot in muskmelons (Singh et al., 2010). The fruit rot symptoms are defined by the existence of light brown to black spots, lesions appearing water-soaked, and irregularly circular in shape, ranging in size from small spots to the decay of the entire fruit (Suwannarach et al., 2019; Zhang et al., 2022a). The infected fruit was covered with masses of mycelium on both the inside and outside (Zhang et al., 2022a). The interior decayed area seemed rotten and was encompassed by tissues that appeared water-soaked (Khuna et al., 2022). Due to the formation of these symptoms, rot disease decreases the fruit’s quality and reduces its visual attractiveness to consumers, resulting in a substantial reduction in its market value (Nuangmek et al., 2023).

The fast rate of global population growth and the increasing trend toward healthier lifestyles have contributed to a significant rise in the demand for muskmelon fruits. As a result, the area of plantations used for growing muskmelon plants has greatly expanded (Khuna et al., 2022). On the contrary, the occurrence and seriousness of certain fungal-based diseases have also risen in instances where plants have been cultivated in sub-optimal areas (Wilkinson et al., 2011; Nuangmek et al., 2019). Fruit rot disease on cantaloupes and muskmelons has been observed in Thailand. However, research on postharvest fruit rot of watermelon and muskmelon in Thailand has been limited. To date, only three *Fusarium* species *F. equiseti* (Nuangmek et al., 2019), *F. incarnatum* (Wonglom and Sunpapao, 2020), and *F. melonis* (Khuna et al., 2022) have been identified as causal agents. Therefore, there is still a need to identify additional causative agents of these diseases in Thailand. In this study, fungal-caused fruit rot disease on muskmelons was observed throughout both postharvest storage periods in 2022 and 2023 (from March to April and from mid-December to January) in Phayao Province, northern Thailand. The disease incidence ranged from 10% to 15% depending on the quantity of fruits (100 fruits per pallet box) contained within each pallet box. Consequently, a substantial portion of the fruit crop was unable to be sold. Therefore, this study aimed to isolate, identify, and assess the pathogenicity of the fungi causing the disease. The obtained fungi were identified by examining their morphological traits along with conducting a multigene phylogenetic analysis. The pathogenicity of the isolated fungi was confirmed through the application of Koch’s postulates. Subsequently, the sensitivity of isolated fungi to some commercial fungicides in solid culture was investigated.

## 2 Materials and methods

### 2.1 Sample collection

Fruit rot disease was observed on muskmelon (*Cucumis melo* L.) fruits throughout the postharvest storage at 26 to 32°C and 65 to 75% relative humidity over a period of 7 to 14 days in Mae Chai District, Phayao Province, northern Thailand in 2022 and 2023 (two periods: March to April and mid-December to January). Ten fruits presenting typical symptoms were shipped to the laboratory within 24 h of being randomly selected and kept in sterile plastic bags. Upon arrival at the laboratory, the fruits exhibiting symptoms were evaluated through a stereo microscope (Nikon H55OS, Tokyo, Japan) and subsequently kept in a plastic container with moist filter paper to encourage sporulation.

### 2.2 Fungal isolation

Samples of fruits were processed in order to isolate fungal causal agents. The method of single conidial isolation described by Choi et al. (1999) was employed to isolate causal fungi from lesions. This process was conducted on 1.0% water agar with an addition of 0.5 mg/L streptomycin under a stereo microscope. The isolated plates were kept in the dark at 25°C for 24–48 h, after which individual germ conidia were moved to potato dextrose agar (PDA; Conda, Madrid, Spain) supplemented with 0.5 mg/L streptomycin. The pure fungal isolates were stored short-term in PDA slants at 4°C and long-term in 20% glycerol at –80°C. The fungal isolates in their pure form were deposited and permanently maintained in a metabolically inactive state at the Sustainable Development of Biological Resources culture collection, Faculty of Science, Chiang Mai University (SDBR-CMU), situated in Chiang Mai Province, Thailand.

### 2.3 Fungal identification

#### 2.3.1 Morphological study

Fungal isolates were examined morphologically employing methodologies outlined by Crous et al. (2021a) and Wang et al. (2019a, b). The characteristics of the colonies, including their colony morphology, pigmentation, and odor, were examined on PDA, oatmeal agar (OA; HiMedia, Maharashtra, India), and synthetic nutrient-poor agar (SNA) after being incubated for seven days in darkness at 25°C. A light microscope (Nikon Eclipse Ni-U, Tokyo, Japan) was employed to conduct micromorphological characteristics. The Tarosoft (R) Image Frame Work software was performed to conduct measurements on at least 50 measurements for each anatomical structure (such as chlamydospores, conidiophores, phialides, and conidia).

#### 2.3.2 DNA extraction, PCR amplification and sequencing

Fungal mycelia cultured for one week on a PDA was utilized for genomic DNA extraction employing the Fungal DNA Extraction Kit (FAVORGEN, Ping-Tung, Taiwan), following the guidelines provided by the manufacturer. Amplification of the calmodulin (*cam*), RNA polymerase second largest subunit (*rpb2*), and translation elongation factor 1-alpha (*tef1-α*) genes was performed through the use of polymerase chain reaction (PCR), with the CAL-228F/CAL-2Rd primers (Carbone and Kohn, 1999), RPB2-5F2/RPB2-7cR primers (O’Donnell et al., 2010), and EF1/EF2 primers (O’Donnell et al., 1998), respectively. The amplification process for the three genes was carried out in individual PCR reactions. A peqSTAR thermal cycler (PEQLAB Ltd., Fareham, UK) was used for the amplification process, which involved an initial denaturation step for 3 min at 95°C, next to 35 cycles of denaturation for 30 s at 95°C, annealing steps for 30 s at 59°C (*cam*), 1 min at 52°C (*rpb2*), and 50 s at 60°C (*tef1-α*), and a final extension step at 72°C for 1 min. A PCR clean-up Gel Extraction NucleoSpin^®^ Gel and a PCR Clean-up Kit (Macherey-Nagel, Düren, Germany) were employed to purify the PCR products in accordance with the manufacturer’s instructions, following which they were examined on a 1% agarose gel electrophoresis. After final purification, direct sequencing was performed on the PCR products. The sequences were automatically determined in the Genetic Analyzer at the 1st Base Company (Kembangan, Malaysia) through sequencing reactions using the PCR primers mentioned earlier.

#### 2.3.3 Sequence alignment and phylogenetic analyses

The BLAST tool, accessible at NCBI (http://blast.ncbi.nlm.nih.gov, accessed on 10 April 2024), was used to conduct similarity searches for the analysis of the *cam*, *rpb2*, and *tef1-α* sequences. The sequences from this study, along with those obtained from previous studies and the GenBank database (with ≥60% query coverage and ≥85–100% sequence similarity), were selected and are listed in **Table 1**. MUSCLE (Edgar, 2004) was utilized for multiple sequence alignment, and BioEdit v. 6.0.7 (Hall, 2004) was performed for any necessary improvements. The combined *cam*, *rpb2*, and *tef1-α* dataset were employed for phylogenetic analysis. The *F. camptoceras* species complex (FCAMSC) was selected to consist of *F. camptoceras* CBS 193.65 and *F. neosemitectum* CBS 115476 as the outgroup. The process for generating a phylogenetic tree involved the utilization of both Bayesian inference (BI) and maximum likelihood (ML) techniques. The ML analysis was performed using RAxML-HPC2 on XSEDE version 8.2.12 (Felsenstein, 1985; Stamatakis, 2006). This analysis utilized 25 categories and 1000 bootstrap replicates with the GTRCAT model of nucleotide substitution, accessed via the CIPRES web portal (Miller et al., 2009). jModeltest version 2.3 (Darriba et al., 2012) was employed to determine the optimal model for nucleotide substitution following the Akaike Information Criterion (AIC) methodology. The BI analysis was determined by Markov Chain Monte Carlo sampling (MCMC) using MrBayes version 3.2 (Ronquist et al., 2012). Six simultaneous Markov chains were performed for four million generations using random beginning trees and trees were sampled every 1000 generations. The run was stopped when the standard deviation of split frequencies reached below 0.01. The first 20% of the generated trees representing the burn-in phase of the analysis were discarded, and the remaining trees were used for calculating Bayesian posterior probabilities (PP) in the majority rule consensus tree. FigTree version 1.4.0 was used to visualize the phylogenetic trees from both ML and BI analyses (Rambaut, 2019).

**Table 1:** Details regarding the sequences utilized in the molecular phylogenetic analysis.

Fungal Taxa	Strain/Isolate	GenBank Accession Number			Reference
		*cam*	*rpb2*	*tef1-α*	
*Fusarium aberrans*	CBS 131385^T^	MN170311	MN170378	MN170445	Xia et al., 2019
*Fusarium aberrans*	CBS 131387	MN170312	MN170379	MN170446	Xia et al., 2019
*Fusarium arcuatisporum*	LC12147^T^	MK289697	MK289739	MK289584	Wang et al., 2019a
*Fusarium arcuatisporum*	LC11639	MK289658	MK289736	MK289586	Wang et al., 2019a
*Fusarium brevicaudatum*	NRRL 43638^T^	GQ505576	GQ505843	GQ505665	O’Donnell et al., 2009
*Fusarium brevicaudatum*	NRRL 43694	GQ505579	GQ505846	GQ505668	O’Donnell et al., 2009
*Fusarium caatingaense*	URM 6779^T^	−	LS398495	LS398466	Santos et al., 2019
*Fusarium caatingaense*	URM 6778	−	LS398494	LS398465	Santos et al., 2019
*Fusarium cateniforme*	CBS 150.25^T^	MN170317	MN170384	MN170451	Xia et al., 2019
*Fusarium citri*	LC6896^T^	MK289668	MK289771	MK289617	Wang et al., 2019a
*Fusarium citrullicola*	SDBR-CMU422^T^	OP020924	OP020928	OP020920	Khuna et al., 2022
*Fusarium citrullicola*	SDBR-CMU423	OP020925	OP020929	OP020921	Khuna et al., 2022
*Fusarium clavum*	CBS 126202^T^	MN170322	MN170389	MN170456	Xia et al., 2019
*Fusarium clavum*	NRRL 34032	GQ505547	GQ505813	GQ505635	O’Donnell et al., 2009
*Fusarium coffeatum*	CBS 635.76^T^	MN120696	MN120736	MN120755	Lombard et al., 2019
*Fusarium coffeatum*	CBS 430.81	MN120697	MN120737	MN120756	Lombard et al., 2019
*Fusarium compactum*	CBS 186.31^ET^	GQ505560	GQ505826	GQ505648	O’Donnell et al., 2009
*Fusarium compactum*	CBS 185.31	GQ505558	GQ505824	GQ505646	O’Donnell et al., 2009
***Fusarium compactum***	**SDBR-CMU483**	**PP758861**	**PP758870**	**PP758879**	**This study**
*Fusarium croceum*	CBS 131777^T^	MN170329	MN170396	MN170463	Xia et al., 2019
*Fusarium croceum*	NRRL 3020	GQ505498	GQ505764	GQ505586	O’Donnell et al., 2009
*Fusarium duofalcatisporum*	CBS 384.94^T^	GQ505564	GQ505830	GQ505652	O’Donnell et al., 2009
*Fusarium duofalcatisporum*	CBS 264.50	GQ505563	GQ505829	GQ505651	O’Donnell et al., 2009
*Fusarium equiseti*	CBS 307.94^NT^	GQ505511	GQ505777	GQ505599	O’Donnell et al., 2009
*Fusarium equiseti*	CBS 245.61	GQ505506	GQ505772	GQ505594	O’Donnell et al., 2009
*Fusarium fasciculatum*	CBS 131382^T^	MN170339	MN170406	MN170473	Xia et al., 2019
*Fusarium fasciculatum*	CBS 131383	MN170340	MN170407	MN170474	Xia et al., 2019
*Fusarium flagelliforme*	CBS 162.57^T^	GQ505557	GQ505823	GQ505645	O’Donnell et al., 2009
*Fusarium flagelliforme*	CBS 259.54	GQ505562	GQ505828	GQ505650	O’Donnell et al., 2009
*Fusarium gracilipes*	NRRL 43635^T^	GQ505573	GQ505840	GQ505662	O’Donnell et al., 2009
*Fusarium guilinense*	LC12160^T^	MK289652	MK289747	MK289594	Wang et al., 2019a
*Fusarium hainanense*	LC11638^T^	MK289657	MK289735	MK289581	Wang et al., 2019a
*Fusarium hainanense*	LC12161	MK289648	MK289748	MK289595	Wang et al., 2019a
*Fusarium humuli*	CQ1039^T^	MK289712	MK289724	MK289570	Wang et al., 2019a
*Fusarium humuli*	CQ1032	MK289710	MK289722	MK289568	Wang et al., 2019a
*Fusarium incarnatum*	CBS 132.73^NT^	MN170342	MN170409	MN170476	Xia et al., 2019
*Fusarium ipomoeae*	LC12165^T^	MK289704	MK289752	MK289599	Wang et al., 2019a
*Fusarium ipomoeae*	LC12166	MK289706	MK289753	MK289600	Wang et al., 2019a
*Fusarium irregulare*	LC7188^T^	MK289680	MK289783	MK289629	Wang et al., 2019a
*Fusarium irregulare*	LC12146	MK289682	MK289738	MK289583	Wang et al., 2019a
*Fusarium jinanense*	LC15878^T^	OQ125271	OQ125521	OQ125131	Han et al., 2023
*Fusarium jinanense*	LPPC076	−	MG788000	MG733180	Lima et al., 2021
*Fusarium jinanense*	LPPC077	−	MG787999	MG733179	Lima et al., 2021
*Fusarium jinanense*	LPPC079	−	MG787996	MN652629	Lima et al., 2021
***Fusarium jinanense***	**SDBR-CMU484**	**PP758862**	**PP758871**	**PP758880**	**This study**
***Fusarium jinanense***	**SDBR-CMU485**	**PP758863**	**PP758872**	**PP758881**	**This study**
***Fusarium jinanense***	**SDBR-CMU486**	**PP758864**	**PP758873**	**PP758882**	**This study**
*Fusarium lacertarum*	NRRL 20423^T^	GQ505505	GQ505771	GQ505593	O’Donnell et al., 2009
*Fusarium lacertarum*	NRRL 36123	GQ505555	GQ505821	GQ505643	O’Donnell et al., 2009
*Fusarium longicaudatum*	CBS 123.73^T^	MN170347	MN170414	MN170481	Xia et al., 2019
*Fusarium longifundum*	CBS 235.79^T^	GQ505561	GQ505827	GQ505649	O’Donnell et al., 2009
*Fusarium luffae*	LC12167^T^	MK289698	MK289754	MK289601	Wang et al., 2019a
*Fusarium luffae*	NRRL 32522	GQ505524	GQ505790	GQ505612	O’Donnell et al., 2009
*Fusarium monophialidicum*	NRRL 54973^T^	MN170349	MN170416	MN170483	Xia et al., 2019
*Fusarium mianyangense*	LC15879^T^	OQ125335	OQ125510	OQ125232	Han et al., 2023
*Fusarium mianyangense*	NRRL 32181	GQ505522	GQ505788	GQ505610	O’Donnell et al., 2009
*Fusarium mianyangense*	NRRL 32182	GQ505523	GQ505789	GQ505611	O’Donnell et al., 2009
***Fusarium mianyangense***	**SDBR-CMU487**	**PP758865**	**PP758874**	**PP758883**	**This study**
***Fusarium mianyangense***	**SDBR-CMU488**	**PP758866**	**PP758875**	**PP758884**	**This study**
*Fusarium mucidum*	CBS 102395^T^	MN170351	MN170418	MN170485	Xia et al., 2019
*Fusarium mucidum*	CBS 102394	MN170350	MN170417	MN170484	Xia et al., 2019
*Fusarium multiceps*	CBS 130386^T^	GQ505577	GQ505844	GQ505666	O’Donnell et al., 2009
*Fusarium nanum*	LC12168^T^	MK289651	MK289755	MK289602	Wang et al., 2019a
*Fusarium nanum*	LC1384	MK289661	MK289764	MK289611	Wang et al., 2019a
*Fusarium neoscirpi*	CBS 610.95^T^	GQ505513	GQ505779	GQ505601	O’Donnell et al., 2009
*Fusarium nothincarnatum*	LC18436^T^	OQ125290	OQ125509	OQ125147	Han et al., 2023
*Fusarium nothincarnatum*	LC18382	OQ125289	OQ125508	OQ125146	Han et al., 2023
*Fusarium pernambucanum*	URM 7559^T^	−	LS398519	LS398489	Santos et al., 2019
*Fusarium pernambucanum*	URM 6801	−	LS398513	LS398483	Santos et al., 2019
*Fusarium persicinum*	CBS 479.83^T^	MN170361	MN170428	MN170495	Xia et al., 2019
*Fusarium persicinum*	CBS 131780	MN170362	MN170429	MN170496	Xia et al., 2019
*Fusarium scirpi*	CBS 447.84^NT^	GQ505566	GQ505832	GQ505654	O’Donnell et al., 2009
*Fusarium scirpi*	CBS 448.84	GQ505504	GQ505770	GQ505592	O’Donnell et al., 2009
*Fusarium serpentinum*	CBS 119880^T^	MN170365	MN170432	MN170499	Xia et al., 2019
*Fusarium sulawesiense*	InaCC F940^T^	LS479422	LS479855	LS479443	Maryani et al., 2019
*Fusarium sulawesiense*	Indo186	LS479426	LS479864	LS479449	Maryani et al., 2019
*Fusarium sulawesiense*	LC18400	OQ125346	OQ125478	OQ125236	Han et al., 2023
*Fusarium sulawesiense*	LC18608	OQ125348	OQ125487	OQ125212	Han et al., 2023
***Fusarium sulawesiense***	**SDBR-CMU489**	**PP758867**	**PP758876**	**PP758885**	**This study**
***Fusarium sulawesiense***	**SDBR-CMU490**	**PP758868**	**PP758877**	**PP758886**	**This study**
***Fusarium sulawesiense***	**SDBR-CMU491**	**PP758869**	**PP758878**	**PP758887**	**This study**
*Fusarium tanahbumbuense*	InaCC F965^T^	LS479432	LS479863	LS479448	Maryani et al., 2019
*Fusarium tanahbumbuense*	NRRL 34005	GQ505541	GQ505807	GQ505629	O’Donnell et al., 2009
*Fusarium toxicum*	CBS 406.86^T^	MN170374	MN170441	MN170508	Xia et al., 2019
*Fusarium toxicum*	CBS 219.63	MN170373	MN170440	MN170507	Xia et al., 2019
*Fusarium weifangense*	LC18333^T^	OQ125276	OQ125515	OQ125107	Han et al., 2023
*Fusarium weifangense*	LC18243	OQ125273	OQ125513	OQ125106	Han et al., 2023
*Fusarium wereldwijsianum*	CBS 148244^T^	MZ921538	MZ921718	MZ921850	Crous et al., 2021b
*Fusarium wereldwijsianum*	CBS 148386	MZ921540	MZ921720	MZ921852	Crous et al., 2021b
*Fusarium camptoceras*	CBS 193.65^ET^	MN170316	MN170383	MN170450	Xia et al., 2019
*Fusarium neosemitectum*	CBS 189.60^T^	MN170355	MN170422	MN170489	Xia et al., 2019

Species designated as neotype, epi-type, and ex-type are represented by the superscript letters “NT”, “ET”, and “T”, respectively. GenBank is missing any sequencing information, represented by the symbol “–”. The fungal isolates and sequences obtained in this study are in bold.

### 2.4 Pathogenicity tests

This experiment utilized conidia obtained from fungal isolates cultured for two weeks on PDA. Healthy commercial muskmelons were washed thoroughly, and then their surfaces were sterilized by soaking them for 5 min in a sterile sodium hypochlorite solution with a concentration of 1.5% (*v/v*). Following that, sterile distilled water was utilized to wash them three times. The fruits were allowed to air dry at room temperature (25 ± 2°C) for a period of 10 min after surface-disinfection (Khuna et al., 2022). Following the air-drying process, aseptic needles were used to create a uniform wound (consisting of 5 pores, each 1 cm deep and 1 mm wide) along the equator of each fruit (Nuangmek et al., 2019). A quantity of 500 µL of a conidial suspension (1 × 10^6^ conidia/mL) from each fungal isolate was applied to the wounded fruits. Accordingly, the control group of wounded fruits received an inoculation of sterile distilled water. Then, the inoculated fruit was kept in an individual sterile plastic container (26 × 35.5 × 20 cm) under 80% relative humidity conditions. The plastic containers were maintained in a growth chamber at a temperature of 25°C under a 12-hour light cycle for a duration of one week. A total of ten replicas were used for each treatment, which was repeated twice under the same condition. The level of disease infections was evaluated using a score of 1–25% (mild), 26–50% (moderate), 51–75% (severe), and 76–100% (very severe) based on the degree of disease infection on the damaged fruit portions (Nuangmek et al., 2023). Confirmation of Koch’s postulates was achieved by re-isolating the fungi through the single-spore isolation method from any lesions that occurred on the inoculated fruits.

### 2.5 Screening of commercial fungicides against *Fusarium* species

Eight commercially available fungicides, inculding benalaxyl-M (4%) + mancozeb (65%) (Fantic M WG^®^, Thailand), captan (Captan 50^®^, Thailand), carbendazim (Dazine^®^, Thailand), copper oxychloride (Copina 85 WP^®^, Thailand), difenoconazole (12.5%) + azoxystrobin (20%) (Ortiva^®^, Thailand), difenoconazole (Score^®^, Thailand), mancozeb (Newthane M-80^®^, Thailand), and metalaxyl (Metalaxyl^®^, Thailand) were examined in this study according the approach indicated through Suwannarach et al. (2015) and Khuna et al. (2023). The fungicides used in this study were available commercially in Thailand and were approved for usage. The *in vitro* applications of benalaxyl-M + mancozeb, captan, carbendazim, copper oxychloride, difenoconazole + azoxystrobin, difenoconazole, mancozeb, and metalaxyl were recommended at dosages of 1380, 750, 750, 1700, 243.75, 187.5, 1200, and 625 ppm, respectively, according to the labels for each fungicide. The final concentration was obtained by preparing each fungicide and adding it to an autoclaved PDA. The test media was added using mycelial plugs (5 mm in diameter) that had been cultivated on PDA for one week in the dark at 25°C. Control did not receive any treatments with fungicide. The plates were maintained in darkness at a temperature of 25°C. Following one week of incubation, the mycelial growth of each isolate was evaluated on in-dividual plates, and a comparison was made between the growth in PDA medium supplemented with fungicides and the growth observed in the control. The calculation of the percentage growth inhibition for each isolate was performed using the formula provided by Achilonu et al. (2023) and Pandey et al. (2024). Each fungal isolate was ranked as sensitive (≥ 50%) or insensitive (< 50%) based on growth inhibition (Yamada et al., 2016; Pandey et al., 2024). Five replications were conducted for each fungicide and fungal isolate. The experiments were independently repeated twice in the same biological conditions.

### 2.6 Statistical analysis

For the normality test, data from the two repeated fungicide sensitivity experiments were analyzed using the Shapiro-Wilk test in SPSS program version 26 at a significance level of *p* < 0.05. The results indicated non-significant findings, so the data from these repeated experiments were assessed for the assumptions of one-way analysis of variance (ANOVA). Duncan’s Multiple Range Test (DMRT) was then employed to identify significant differences at *p* ≤ 0.05.

## 3 Results

### 3.1 Sample collection and disease symptoms

**Figure 1:**
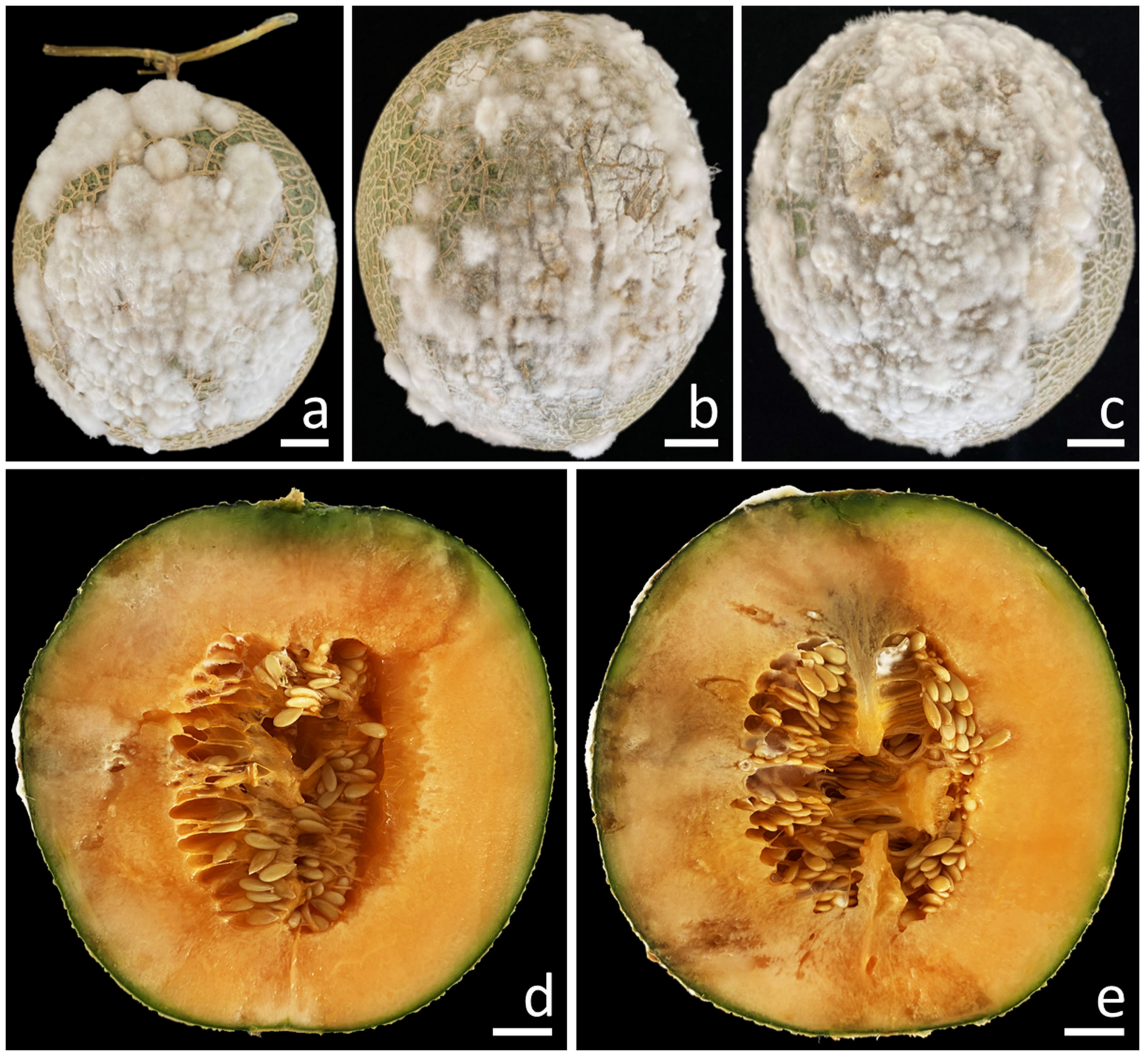
Symptoms of fruit rot in muskmelon during the postharvest storage period **(A–C)**. A cross-sectional view of the infected muskmelon fruits reveals the areas of internal decay **(D, E)**. Scale bars: **(A–C)** = 20 mm; **(D, E)** = 15 mm.

A total of 10 samples of fruit rot on muskmelon were taken from postharvest storage pallet boxes situated in Phayao Province, northern Thailand. The initial appearance of the symptoms was in the middle and base of the muskmelon, displaying as brown spots encircled by a bruised edge. Ultimately, advanced lesions became covered with white mycelial masses (**Figures 1A–C**). The lesions on the muskmelon fruit eventually expanded and merged, covering the entire fruit, resulting in a bruised, ruptured, and decayed appearance for infected fruits. The inner portion appeared distinctly rotten and was encompassed by tissue soaked in water (**Figures 1D, E**).

### 3.2 Fungal isolation

A total of nine fungal isolates (CMU483 to CMU491) were derived from the collected muskmelons displaying characteristic rot symptoms. All the fungal isolates were stored for short periods of time in potato dextrose agar (PDA) slants at 4°C and for longer periods of time in 20% glycerol at –80°C. The fungal isolates were all submitted and maintained in a permanently inactive condition at the Sustainable Development of Biological Resources culture collection, Faculty of Science, Chiang Mai University (SDBR-CMU), located in Chiang Mai Province, Thailand. They were assigned accession codes ranging from SDBR-CMU483 to SDBR-CMU491, respectively.

### 3.3 Morphological study

Three different types of agar media, namely PDA, oatmeal agar (OA), and synthetic nutrient-poor agar (SNA), were employed to examine fungal colonies of each isolate. Following one week of incubation at 25°C, OA was demonstrated to be the optimal medium as it exhibited the largest colony diameter among all fungal isolates. In all agar media, each of the nine fungal isolates exhibited the formation of conidiophores, chlamydospores, phialides, and conidia. Upon examination of their morphological traits, all the fungal isolates were initially classified as members of the genus *Fusarium* (Wang et al., 2019a, 2022b; Xia et al., 2019; Crous et al., 2021a). The findings derived from morphological examination of the fungal colony and micromorphological characteristics indicated that the isolate SDBR-CMU484 exhibited similarities with isolates SDBR-CMU485 and SDBR-CMU486, while the isolate SDBR-CMU487 was related to the isolate SDBR-CMU488. Additionally, isolates SDBR-CMU489, SDBR-CMU490, and SDBR-CMU491 showed similarities.

### 3.4 Phylogenetic analysis

**Figure 2:**
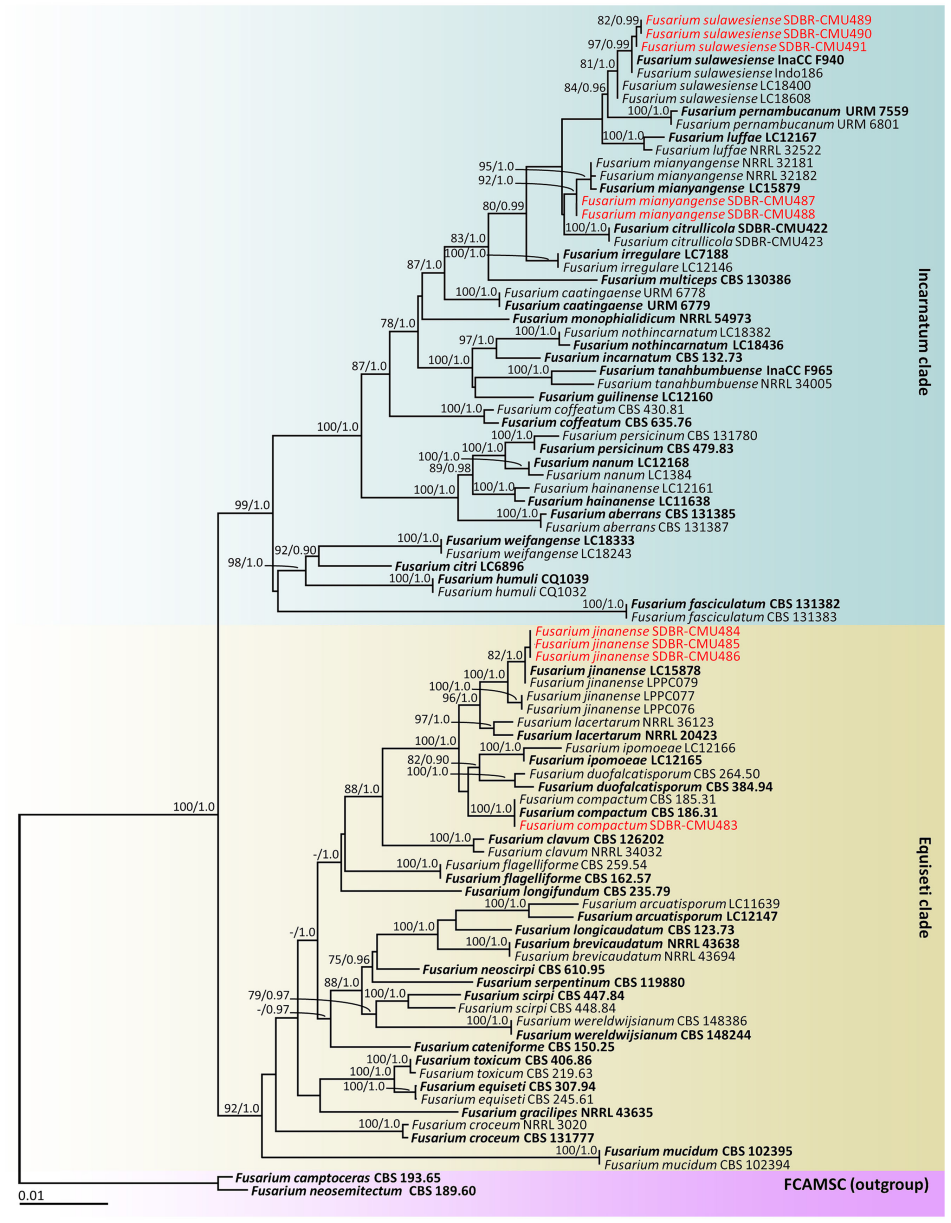
Phylogram generated through maximum likelihood analysis of a combination of *cam*, *rpb2*, and *tef1-α* genes of 91 sequences. *Fusarium camptoceras* CBS 193.65 and *F. neosemitectum* CBS 115476 were employed as outgroups. Bootstrap values ≥ 75% ML (left) and Bayesian posterior probabilities ≥ 0.90 (right) are displayed above nodes. The expected number of nucleotide substitutions per site are indicated by the scale bar. Red represents the fungus species’ sequences found in the current study. Type species are in bold.

According to the BLAST results, all fungal isolates were identified as members of the *F. incarnatum-equiseti* species complex. The combined *cam*, *rpb2*, and *tef1-α* sequences dataset consists of 91 taxa, and the aligned dataset includes 2132 characters comprising gaps (*cam*: 1–604, *rpb2*: 605–1484, and *tef1-α*: 1485–2132). The best scoring RAxML tree was established with a final ML optimization likelihood value of –9911.6997. Accordingly, the matrix contained 625 distinct alignment patterns with 7.67% undetermined characters or gaps. The estimated base frequencies were found to be: A = 0.2309, C = 0.2899, G = 0.2149, and T = 0.2643; substitution rates AC = 0.7528, AG = 3.1066, AT = 1.3768, CG = 0.8323, CT = 5.5758, and GT = 1.0000. The values of the gamma distribution shape parameter alpha and the Tree-Length were 0.2528 and 0.6387, respectively. Additionally, BI analysis yielded a final average standard deviation of 0.005188 for the split frequencies at the end of all MCMC generations. Regarding topology, the phylograms generated from the ML and BI analyses exhibited similarity (data not displayed). Consequently, the phylogenetic tree obtained from the ML analysis was selected and is displayed in **Figure 2**.

Our phylogenetic tree was generated in a concordant approach and is corroborated by earlier investigations (Wang et al., 2019a, 2022b; Xia et al., 2019; Crous et al., 2021a; Khuna et al., 2022; Han et al., 2023). A phylogram assigned the two fungal isolates (SDBR-CMU487 and SDBR-CMU488) and three fungal isolates (SDBR-CMU489, SDBR-CMU490, and SDBR-CMU491) in this study within the same clade of *F. mianyangense* and *F. sulawesiense*, which consisted of the type species LC15879 and InaCC F940, respectively, within the Incarnatum clade. *Fusarium mianyangense* appeared as a closely related taxon to *F. citrullicola*, while *F. sulawesiense* formed a sister taxon to *F. pernambucanum* with high statistical support (84% BS and 0.96 PP). Therefore, both fungal isolates (SDBR-CMU487 and SDBR-CMU488) and three fungal isolates (SDBR-CMU489, SDBR-CMU490, and SDBR-CMU491) were identified as *F. mianyangense* and *F. sulawesiense*, respectively. Additionally, one fungal isolate (SDBR-CMU483) and three fungal isolates (SDBR-CMU484, SDBR-CMU485, and SDBR-CMU486) obtained in this study were also positioned within the *F. compactum* and *F. jinanense*, which included the type species CBS 186.31 and LC15878, respectively, in the *Equiseti* clade. *Fusarium compactum* constituted a species that showed phylogenetic relation to both *F. duofalcatisporum* and *F. ipomoeae*. While *F. jinanense* constituted a species that exhibited strong statistical support (96% BS and 1.0 PP) for its phylogenetic relation to *F. lacertarum*. Thus, this one fungal isolate (SDBR-CMU483) and three fungal isolates (SDBR-CMU484, SDBR-CMU485, and SDBR-CMU486) were recognized as *F. compactum* and *F. jinanense*, respectively.

### 3.5 Morphological description

#### 3.5.1 *Fusarium compactum* (Wollenw.) Raillo, fungi of the genus *Fusarium*, 180 (1950)

**Figure 3:**
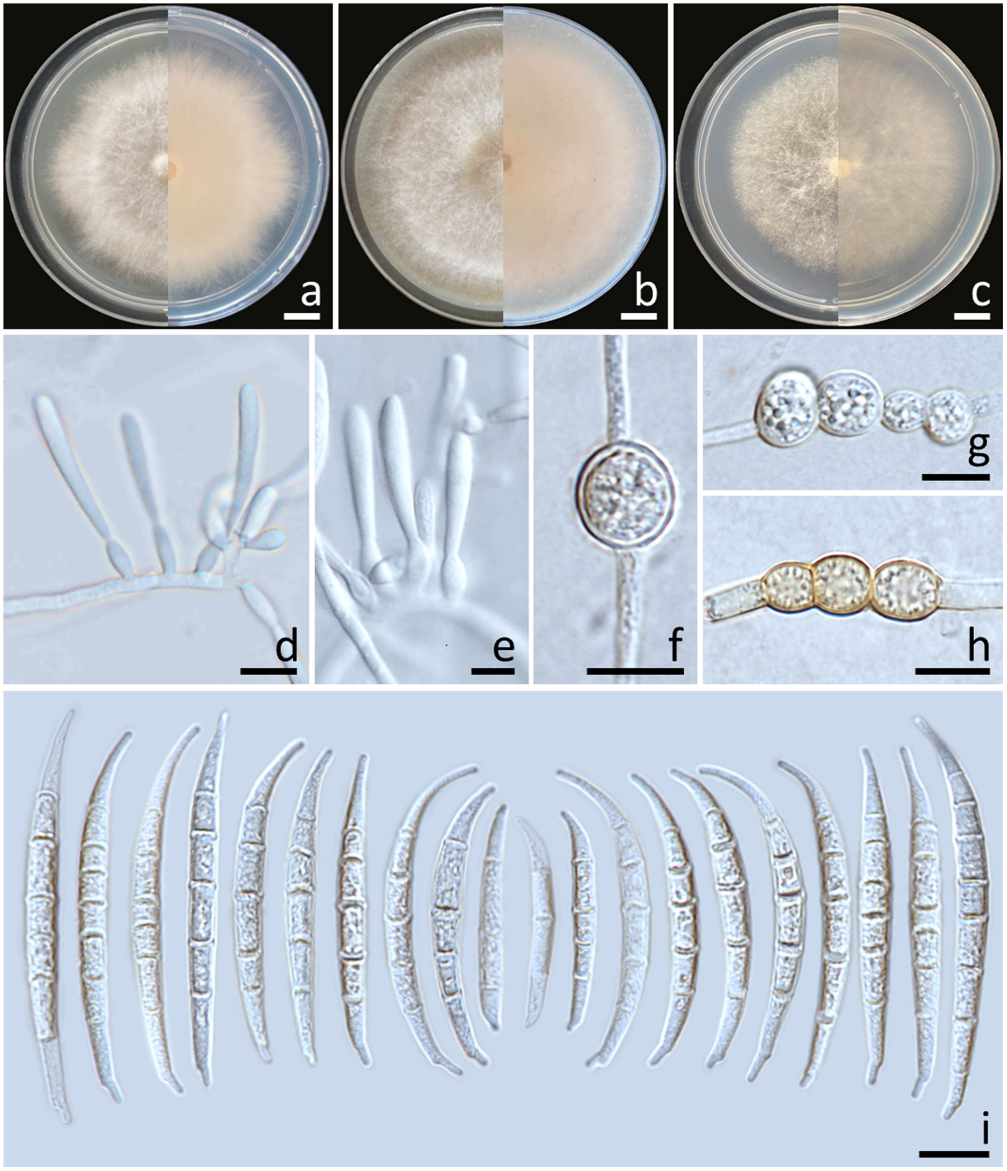
*Fusarium compactum* (SDBR-CMU483). Colony on potato dextrose agar **(A)**, oatmeal agar **(B)** and synthetic nutrient-poor agar **(C)** (left, surface view and right, reverse view) after incubation for one week at 25°C. Phialides on mycelium **(D, E)**. Chlamydospores **(F–H)**. Conidia **(I)**. Scale bars: **(A–C)** = 10 mm; **(D–I)** = 10 µm.

Notes: The morphological characteristics of the *F. compactum* fungal isolates obtained in this study were consistent with previous descriptions of *F. compactum* (Raillo, 1950; Leslie and Summerell, 2006). Phylogenetically, *F. compactum* formed a species that was phylogenetically related to *F. duofalcatisporum* and *F. ipomoeae*. However, the growth of *F. compactum* exhibited slower growth compared to *F. duofalcatisporum* on PDA (75–82 mm) and *F. ipomoeae* on PDA (53–57 mm) and SNA (51–56 mm) after one week of incubation at 25°C (Wang et al., 2019a; Xia et al., 2019). Additionally, *F. compactum* grew on OA faster than *F. ipomoeae* (52–63 mm) (Wang et al., 2019a). Based on micromorphology, *F. duofalcatisporum* could be distinguished from *F. compactum* by its shorter conidiophores (9–16 μm) (Xia et al., 2019). In addition, the absence of chlamydospores is another way to distinguish *F. ipomoeae* from *F. compactum* (Wang et al., 2019a).

Colonies diameter after incubation at 25°C for one week on PDA, OA, and SNA grew to 35.0–43.0, >85.0, and 30.0–38.0 mm in diameter, respectively (**Figure 3**). Colonies on PDA were yellowish white in the center, white at the margins, flat with entire edges; reverse pale yellow. Colonies on OA were greyish yellow in the center, white at the margin, dense aerial mycelia, slightly raised with entire edges; reverse greyish orange. Colonies on SNA were white, flat with entire edges; reverse white. No pigment or odor was present. Sporodochia were absent on all agar media. Conidiophores developed on aerial mycelium, 15–90 × 2.6–4.1 µm, sympodial or irregularly branched, bearing terminal or lateral phialides. Phialides were monophialidic, subulate to sub-cylindrical, hyaline, smooth and thin-walled, 7.6–30 × 2.6–4.5 µm. Chlamydospores were abundant, globose, ellipsoid, intercalarily or terminal, smooth-walled, solitary, in chains or clusters, hyaline to pale yellow with age, 5.9–19.1 × 6–15.3 µm. Conidia were hyaline, thick-walled, strongly curved, elongated apical cell, well-developed to slightly elongated foot-shaped basal cell, 3–8-septate, 13.3–66.2 × 2.6–4.9 μm (av. ± SD: 39.3 ± 13.3 × 3.8 ± 0.5 µm).

#### 3.5.2 *Fusarium jinanense* S.L. Han, M.M. Wang & L. Cai, *Stud. Mycol.* 104: 131 (2023)

**Figure 4:**
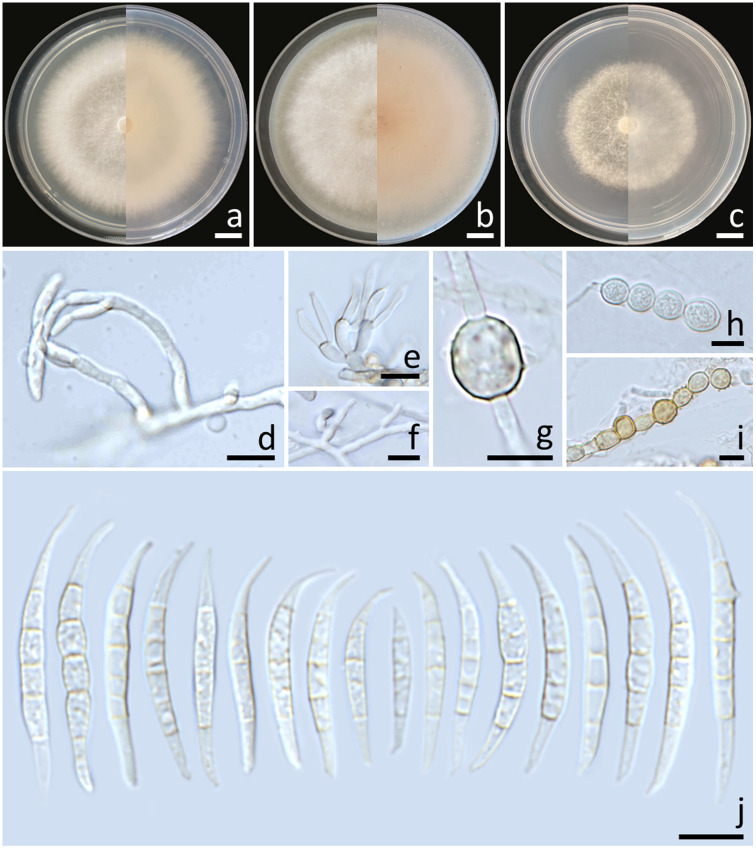
*Fusarium jinanense* (SDBR-CMU484). Colony on potato dextrose agar **(A)**, oatmeal agar **(B)** and synthetic nutrient-poor agar **(C)** (left, surface view and right, reverse view) after incubation for one week at 25°C. Conidiophores and phialides **(D–F)**. Chlamydospores **(G–I)**. Conidia **(J)**. Scale bars: **(A–C)** = 10 mm; **(D–I)** = 10 µm.

Notes: Morphologically, the fungal isolates of *F. jinanense* obtained in this study closely resembled the descriptions of *F. jinanense* provided in previous studies (Han et al., 2023). Phylogenetically, *F. jinanense* is closely related to *F. lacertarum*. Nonetheless, the shorter conidiophores (up to 7.0 µm long) and smaller phialides (2.5–4.0 × 1.0–1.5 μm) of *F. lacertarum* help to distinguish it from *F. jinanense* (Subrahmanyam, 1983).

Colonies diameter after incubation at 25°C for one week on PDA, OA, and SNA grew to 69.0–77.0, >85.0, and 61.0–67.0 mm in diameter, respectively (**Figure 4**). Colonies on PDA were white, flat with entire edges; reverse yellowish white. Colonies on OA were white, flat with entire edges; reverse orange white. Colonies on SNA were white, flat with entire edges; reverse white. No pigment or odor was present. Sporodochia were absent on all agar media. Conidiophores developed on arerial mycelium, 9.6–154.1 × 2.5–5.6 μm, irregularly branched. Phialides were mono- and polyphialidic, subulate to sub-cylindrical, smooth, thin-walled, 5.8–12.1 × 2.3–4 μm. Chlamydospores were abundant, globose, hyaline to light yellow with age, smooth or rough-walled, intercalary or terminal, solitary, in pairs or forming long chains, 4.9–13.4 × 4.9–12.7 μm. Conidia falcate, curved dorsoventrally, tapering towards both ends, elongated or whip-like curved apical cell, well-developed foot-shaped basal cell, hyaline, smooth, thin-walled, 3–7-septate, 14–49.7 × 2.7–5.9 μm (av. ± SD: 32.7 ± 6.2 × 4.1 ± 0.6 μm).

#### 3.5.3 *Fusarium mianyangense* S.L. Han, M.M. Wang & L. Cai, *Stud. Mycol.* 104: 131 (2023)

**Figure 5:**
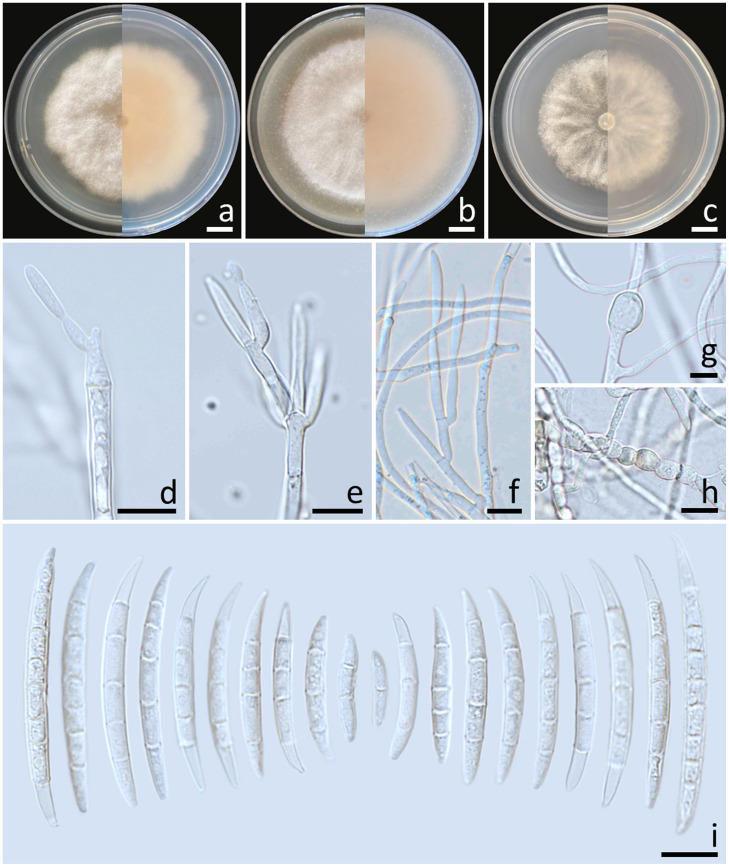
*Fusarium mianyangense* (SDBR-CMU487). Colony on potato dextrose agar **(A)**, oatmeal agar **(B)** and synthetic nutrient-poor agar **(C)** (left, surface view and right, reverse view) after incubation for one week at 25°C. Conidiophores and phialides **(D–F)**. Chlamydospores **(G, H)**. Conidia **(I)**. Scale bars: **(A–C)** = 10 mm; **(D–I)** = 10 µm.

Notes: The fungal isolates of *F. mianyangense* obtained in this study exhibited morphological characteristics consistent with the earlier descriptions of *F. mianyangense* (Han et al., 2023). However, the number of septa conidia in *F. mianyangense* observed in this study (1–8-septate) was more than those reported in the result of Han et al. (2023) (3–5-septate). Phylogenetically, *F. mianyangense* is closely related to *F. citrullicola*. Nevertheless, the growth of *F. mianyangense* exhibited slower growth compared to *F. citrullicola* on PDA (68.0–74.5 mm), but faster than *F. citrullicola* on OA (75.0–85.0 mm) over a one-week period at 25°C (Khuna et al., 2022). Micromorphology, *F. citrullicola* could be distinguished from *F. mianyangense* by its shorter conidia (8.0–39.0 μm) (Khuna et al., 2022). In addition, the number of septa conidia of *F. citrullicola* (1–5-septate) was less than that of *F. mianyangense* (1–8-septate).

Colonies diameter after incubation at 25°C for one week on PDA, OA, and SNA grew to 57.0–62.0, >85.0, and 47.0–53.0 mm in diameter, respectively (**Figure 5**). Colonies on PDA were yellowish orange in the center, reddish white at the margins, raised with entire edges; reverse reddish white. Colonies on OA were white, dense aerial mycelia, umbilicate with entire edges; reverse greyish orange. Colonies on SNA were white, raised with undulate entire edges; reverse white. No pigment or odor was present. Sporodochia were absent on all agar media. Conidiophores developed on arerial mycelium, 6.4–118.5 × 2.2–4.1 μm, irregularly or verticillately branched. Phialides were mono- and polyphialidic, subulate to sub-cylindrical, smooth, thin-walled, 8.6–29.2 × 1.9–7.7 μm. Chlamydospores were abundant, globose to ellipsoidal, hyaline to pale yellow with age, smooth, intercalary or terminal, solitary or forming long chains, 5.6–25.5 × 5.4–21.8 μm. Conidia were falcate, hyaline, smooth, thin-walled, unequally curved, pointed to blunt apical cell, poorly-developed foot-shaped basal cell, 1–8-septate, 13.6–57.7 × 2.1–4.5 μm (av. ± SD: 27.8 ± 12.1 × 3.1 ± 0.5 μm).

#### 3.5.4 *Fusarium sulawesiense* Maryani, Sand.-Den., L. Lombard, Kema & Crous [as ‘*sulawense*’], *Persoonia* 43: 65 (2019)

**Figure 6:**
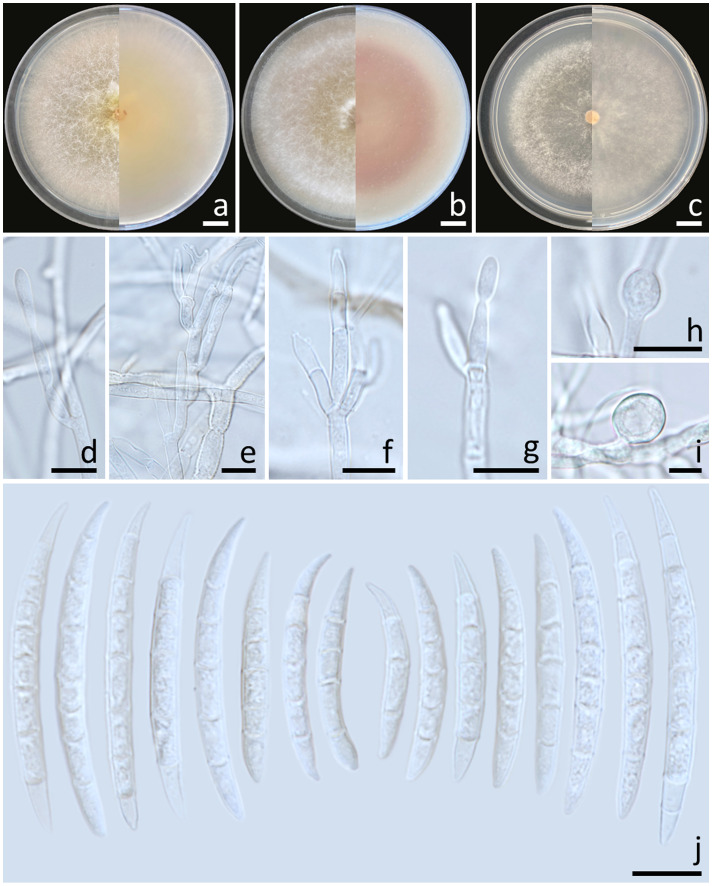
*Fusarium sulawesiense* (SDBR-CMU489). Colony on potato dextrose agar **(A)**, oatmeal agar **(B)** and synthetic nutrient-poor agar **(C)** (left, surface view and right, reverse view) after incubation for one week at 25°C. Conidiophores and phialides **(D–G)**. Chlamydospores **(H, I)**. Conidia **(J)**. Scale bars: **(A–C)** = 10 mm; **(D–J)** = 10 µm.

Notes: Morphologically, the fungal isolates of *F. sulawesiense* obtained in this study were consistent to those obtained from previous studies of the species (Maryani et al., 2019; Yi et al., 2022). Phylogenetically, *F. sulawesiense* is closely related to *F. pernambucanum*. However, *F. pernambucanum* could be distinguished from *F. sulawesiense* by its longer phialides (up to 62.5 μm) and smaller chlamydospores (5–8 μm) (Santos et al., 2019).

Colonies diameter after incubation at 25°C for one week on PDA, OA, and SNA grew to 83.0–85.0, >85.0, and 77.0–81.0 mm in diameter, respectively (**Figure 6**). Colonies on PDA were greyish yellow in the center, orange white at the margins, raised with entire edges; reverse light yellow. Colonies on OA were white, raised with entire edges; reverse greyish orange. Colonies on SNA were pastel yellow in the center, white at the margins, flat with entire edges; reverse pale yellow. No pigment or odor was present. Sporodochia were absent on all agar media. Conidiophores developed on arerial mycelium, 8.9–100 × 2.5–5 μm, septate, irregularly or verticillately branched. Phialides were mono- and polyphialidic, subulate to sub-cylindrical, smooth, thin-walled, formed singly, laterally or terminally, sometimes proliferating percurrently, 9.5–25.7 × 2–4.6 μm. Chlamydospores were hyaline, globose to ellipsoidal, solitary, intercalary or terminal, 11.8–28 × 7.5–23 μm. Conidia were formed on both mono- and polyphialides, falcate, curved dorsiventrally, hyaline, pointed apical cell, indistinct or papillate basal cells, 3–8-septate, 18.1–59.5 × 3–6 μm (av. ± SD: 34.0 ± 8.2 × 4.5 ± 0.8 μm).

### 3.6 Pathogenicity test

**Figure 7:**
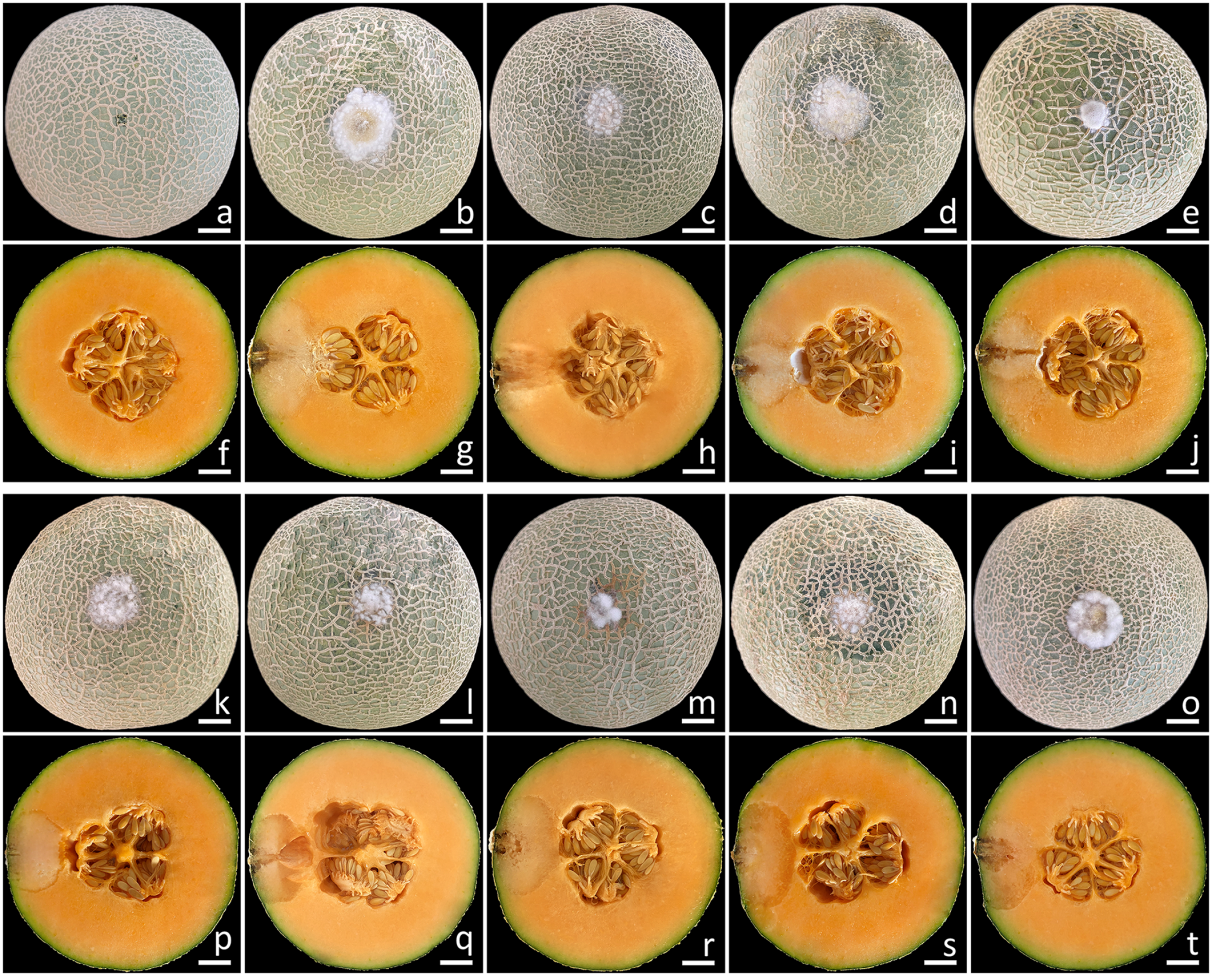
Pathogenicity test using *F. compactum* (SDBR-CMU483), *F. jinanense* (SDBR-CMU484, SDBR-CMU485, and SDBR-CMU486), *F. mianyangense* (SDBR-CMU487 and SDBR-CMU488), and *F. sulawesiense* (SDBR-CMU489, SDBR-CMU490, and SDBR-CMU491) on muskmelon fruits after one week of inoculation. Control fruit inoculated with sterile water **(A, F)**. Disease symptoms after inoculation with isolate SDBR-CMU483 **(B, G)**, SDBR-CMU484 **(C, H)**, SDBR-CMU485 **(D, I)**, SDBR-CMU486 **(E, J)**, SDBR-CMU487 **(K, P)**, SDBR-CMU488 **(L, Q)**, SDBR-CMU489 **(M, R)**, SDBR-CMU490 **(N, S)**, and SDBR-CMU491 **(O, T)**. Scale bars = 20 mm.

This experiment employed the conidia from all fungal isolates. The initial symptoms appeared on the muskmelon fruits two days after being inoculated. In the beginning, the fruits exhibited small spots that ranged in color from yellowish-brown to light brown. Subsequently, the lesions on the fruits rapidly expanded, and some fruits exhibited greenish bruised areas, which were surrounded by white mycelia encompassing each lesion. Following a week of incubation, the sizes of the lesions on the inoculated fruits ranged from 2.0 to 3.0 cm in diameter (**Figure 7**), and the muskmelons displayed mild infection (disease scores of 5–15%), as indicated by the presence of rot symptoms. A cross-sectional examination indicated that the internal lesion area seemed to be decomposing and was surrounded by tissue soaked with water (**Figures 7F–J, P–T**). The internal lesions on the fruits had diameters ranging from 3.5 to 4.5 cm. The lesions subsequently expanded and developed necrosis within 14 to 16 days on muskmelon samples, which were categorized as moderate to severe infections (disease scores of 30–70%). A very severe infection (disease scores of 80–85%) was observed after three weeks of incubation. In the end, the fruits were entirely soft and rotten. These disease symptoms resembled those found throughout the postharvest storage period. Nevertheless, the wounded fruits treated with sterile distilled water did not exhibit any disease symptoms (**Figures 7A, F**). The fungi from each inoculated tissue were consistently re-isolated before being cultivated on PDA to satisfy Koch’s postulates. The re-isolated fungi were identified as *F. compactum*, *F. jinanense*, *F. mianyangense*, and *F. sulawesiense*.

### 3.7 Reactions of commercial fungicides against *Fusarium* pathogens

The effects of fungicides at recommended dosages on the mycelial growth of *Fusarium* species obtained in this study were reported in terms of the percentage of mycelial inhibition, as shown in **Table 2**. The results revealed that the inhibition values varied among different fungicides, fungal species, and fungal isolates. Data on the percentage of mycelial inhibition for each fungal isolate, related to the fungicides, passed the normality test (Shapiro-Wilk test, *p*-value < 0.001), thereby assuming normal distributions. Therefore, ANOVA followed by DMRT (*p* ≤ 0.05) was used to identify significant differences. According to the findings, COOX (copper oxychloride) significantly outperformed other fungicides in terms of the percentage of mycelial inhibition of all isolates of *F. compactum*, *F. mianyangense*, and *F. sulawesiense*. For *F. jinanense* SDBR-CMU484 and SDBR-CMU485, DI (difenoconazole) showed the highest percentage of mycelial inhibition, whereas DI+A (difenoconazole + azoxystrobin) showed the highest percentage of mycelial inhibition for *F. jinanense* SDBR-CMU486. Additionally, the inhibition values ≥ 50% and < 50% were classified as sensitive and insensitive reactions, respectively. All isolates of *F. compactum*, *F. jinanense*, *F. mianyangense*, and *F. sulawesiense* were sensitive to COOX. Additionally, all isolates of *F. jinanense* and *F. mianyangense* were sensitive to DI+A and DI. The sensitivity to CA (captan) was found only in *F. jinanense*. On the other hand, all fungal isolates showed insensitivity to B+M (benalaxyl-M + mancozeb), CAR (carbendazim), MA (mancozeb), and ME (metalaxyl). The insensitivity to CA was found in *F. compactum*, *F. mianyangense*, and *F. sulawesiense*. Furthermore, the results indicated that *F. compactum* and *F. sulawesiense* were insensitive to DI+A and DI.

**Table 2:** Reactions of 9 isolates of *Fusarium* species against synthetic fungicides.

*Fusarium* species/Isolate	Percentage of mycelial inhibition (%)*								Reactions
	B+M	CA	CAR	COOX	DI+A	DI	MA	ME	
*F. compactum*/ SDBR-CMU483	37.45 ± 1.95 b	31.63 ± 2.63 c	15.82 ± 1.95 f	62.76 ± 1.95 a	11.22 ± 2.63 g	22.96 ± 2.57 d	38.78 ± 1.67 b	19.90 ± 1.95 e	Sensitive to COOX
*F. jinanense*/ SDBR-CMU484	14.51 ± 1.28 f	58.82 ± 1.97 c	36.08 ± 1.50 d	73.33 ± 1.81 b	73.33 ± 1.28 b	76.47 ± 1.28 a	20.78 ± 1.25 e	10.20 ± 1.50 g	Sensitive to CA, COOX, DI+A, and DI
*F. jinanense*/ SDBR-CMU485	24.79 ± 2.57 f	59.20 ± 1.99 c	37.81 ± 3.40 d	60.48 ± 1.64 bc	62.19 ± 1.62 b	67.66 ± 1.91 a	14.23 ± 2.57 g	30.85 ± 1.91 e	Sensitive to CA, COOX, DI+A, and DI
*F. jinanense*/ SDBR-CMU486	30.78 ± 1.98 f	58.55 ± 1.69 d	37.82 ± 1.69 e	64.25 ± 3.11 bc	70.98 ± 1.28 a	63.21 ± 1.98 c	25.89 ± 1.98 g	9.33 ± 1.98 h	Sensitive to CA, COOX, DI+A, and DI
*F. mianyangense/* SDBR-CMU487	43.26 ± 2.40 d	33.02 ± 1.52 ef	32.09 ± 2.63 f	75.81 ± 1.52 a	64.19 ± 0.93 c	65.12 ± 1.78 bc	14.42 ± 1.52 g	33.95 ± 1.52 ef	Sensitive to COOX, DI+A, and DI
*F. mianyangense/* SDBR-CMU488	26.25 ± 1.60 f	38.33 ± 1.92 e	26.25 ± 1.60 f	78.75 ± 2.10 a	57.50 ± 2.15 bc	58.75 ± 2.10 b	13.25 ± 1.62 g	40.42 ± 1.60 d	Sensitive to COOX, DI+A, and DI
*F. sulawesiense*/ SDBR-CMU489	5.60 ± 1.65 fg	46.63 ± 1.62 b	6.03 ± 2.23 f	74.57 ± 1.65 a	29.74 ± 2.17 e	33.62 ± 2.23 cd	34.91 ± 0.86 c	4.74 ± 1.65 g	Sensitive to COOX
*F. sulawesiense*/ SDBR-CMU490	9.64 ± 2.09 g	25.00 ± 2.45 d	18.59 ± 2.45 f	66.67 ± 2.09 a	21.41 ± 1.48 e	31.28 ± 1.48 c	43.23 ± 2.96 b	5.77 ± 2.45 h	Sensitive to COOX
*F. sulawesiense*/ SDBR-CMU491	4.95 ± 2.29 f	47.50 ± 1.14 b	11.37 ± 1.90 e	57.76 ± 1.00 a	17.82 ± 1.14 d	23.27 ± 0.99 c	11.39 ± 1.90 e	4.46 ± 1.90 f	Sensitive to COOX

*Results are means ± SD of five replicates with repeated twice. Data with different letters within the same role indicate a significant difference at *p* ≤ 0.05 according to Duncan’s multiple range test. B+M, benalaxyl-M + mancozeb; CA, captan; CAR, carbendazim; COOX, copper oxychloride; DI+A, difenoconazole + azoxystrobin; DI, difenoconazole; MA, mancozeb; ME, metalaxyl.

## 4 Discussion

*Fusarium* species are widely recognized as one of the most significant genera since they are known to cause major diseases in numerous economically valuable crops cultivated worldwide, including muskmelons (Ajmal et al., 2023; Ekwomadu and Mwanza, 2023). Conventionally, the primary approaches used to identify *Fusarium* species have been their macromorphological and micromorphological features (Leslie and Summerell, 2006; Rahjoo et al., 2008; Crous et al., 2021a). However, morphological features are insufficient for distinguishing closely related *Fusarium* species because of the extensive range of morphological variations (Leslie and Summerell, 2006; Crous et al., 2021a). Therefore, molecular techniques are crucial for accurately identifying *Fusarium* at the species level. Researchers have utilized ribosomal DNA [the internal transcribed spacer (ITS) and the large subunit (LSU) regions] and protein-coding genes [*β*-tubulin (*tub2*), *cam*, *tef1-α*, and RNA polymerase largest subunit (*rpb1* and *rpb2*)] as powerful tools to identify *Fusarium* species (Geiser et al., 2004; Nitschke et al., 2009; O’Donnell et al., 2010; Maryani et al., 2019; Wang et al., 2019a; Crous et al., 2021a; Jedidi et al., 2021). However, the accurate identification of *Fusarium* species at the species level remained unresolved when depending solely on the ribosomal DNA gene (Balajee et al., 2009; O’Donnell et al., 2015). Consequently, accurate identification of *Fusarium* species, particularly within the *F. incarnatum-equiseti* species complex, which exhibits a high level of cryptic speciation, is achieved through the combination of morphological features with multi-gene molecular phylogeny (O’Donnell et al., 2010; Maryani et al., 2019; Santos et al., 2019; Wang et al., 2019a, 2022b; Xia et al., 2019; Crous et al., 2021a). In this study, one isolate of *F. compactum* (SDBR-CMU483), three isolates of *F. jinanense* (SDBR-CMU484, SDBR-CMU485, and SDBR-CMU486), two isolates of *F. mianyangense* (SDBR-CMU487 and SDBR-CMU488), and three isolates of *F. sulawesiense* (SDBR-CMU489, SDBR-CMU490, and SDBR-CMU491), were obtained from the rot lesions of muskmelon fruits from northern Thailand. The identification of these fungal species followed methods similar to those employed in the identification of *Fusarium*, which involve combining phylogenetic analysis of multiple genes with their morphological traits (Santos et al., 2019; Wang et al., 2019a, 2022b; Crous et al., 2021a).

Koch’s postulates were fulfilled by conducting pathogenicity tests on all isolates of *F. compactum*, *F. jinanense*, *F. mianyangense*, and *F. sulawesiense*. The findings demonstrate that fruit rot disease in muskmelons, caused by these four *Fusarium* species identified in this study, resembles that caused by previously identified fungal pathogens affecting muskmelons worldwide (Kobayashi et al., 2004; Kwon et al., 2009; Pornsuriya and Chitphithak, 2018; Mirtalebi et al., 2019; Suwannarach et al., 2019; Broge et al., 2020; Lima et al., 2021; Das et al., 2023; Huo et al., 2023; de Almeida Nogueira et al., 2023; Namisy et al., 2023). Our findings are in accordance with the findings of several previous studies, which have demonstrated the economic significance of *Fusarium* as a plant pathogen (Ekwomadu and Mwanza, 2023; Zakaria, 2023). Accordingly, several species within the *F. incarnatum-equiseti* species complex have been documented as the cause of fruit rot disease in cantaloupe, melons and muskmelons around the world. For instance, *F. equiseti* caused fruit rot disease on oriental melon and cantaloupe specimens collected in Korea (Kim and Kim, 2004), Thailand (Nuangmek et al., 2019), and China (Li et al., 2019). Postharvest fruit rot on muskmelons and oriental melons caused by *F. incarnatum* has been reported in Thailand (Wonglom and Sunpapao, 2020) and in Korea (Kim and Kim, 2004), respectively. In 2022, *F. melonis* has been reported as a causal agent of muskmelon fruit rot in Thailand (Khuna et al., 2022). In Brazil, *F. jinanense*, *F. pernambucanum* and *F. sulawesiense* caused posthinvest fruit rot on melons (Araújo et al., 2021; Lima et al., 2021; de Freitas et al., 2024). In China, *F. incarnatum*, *F. luffae*, *F. nanum*, *F. pernambucanum*, and *F. sulawesiense* have been identified as causing fruit rot on muskmelons (Wang et al., 2019b; Zhang et al., 2022a, b, Zhang et al., 2023; Liu et al., 2023). Furthermore, fruit rot on muskmelons has been attributed to various *Fusarium* species from different complexes, including the *F. fujikuroi* species complex (such as *F. annulatum*, *F. moniliforme*, and *F. proliferatum*), the *F. oxysporum* species complex (including *F. kalimantanense* and *F. oxysporum*), the *F. sambucinum* species complex (comprising *F. asiaticum*, *F. graminearum*, and *F. sambucinum*), and the *F. solani* species complex (including *F. falciforme* and *F. solani*) (Champaco and Martyn, 1993; Kim and Kim, 2004; Araújo et al., 2021; Hao et al., 2021; Parra et al., 2022; de Almeida Nogueira et al., 2023).

Various fungicides have been employed to control fungal-caused plant diseases. The insensitivity of plant pathogenic fungi to fungicides indicates their capability to resist them. The efficacy of fungicides in both sensitive and insensitive effects on the *in vitro* mycelial growth of plant pathogenic fungi, especially *Fusarium* species, has been documented in several studies (Mande, 2003; Orina et al., 2020; Zhao and Huang, 2023; Pandey et al., 2024). In this study, the insensitivity of *Fusarium* species to fungicides varied among different fungicides, species, and isolates. These results were consistent with previous studies, which reported that the insensitivity of *Fusarium* species to fungicides varies based on the type and dosage of each fungicide, fungal species, and fungal isolates (Orina et al., 2020; Maniçoba et al., 2023; Zhao and Huang, 2023; Pandey et al., 2024). For example, Baria and Rakholiya (2020) who found that carbendazim, copper oxychloride, and mancozeb were highly sensitive to *F. musae* causing banana fruit rot disease. All isolates of *F. compactum*, *F. jinanense*, *F. mianyangense*, and *F. sulawesiense* obtained in this study were sensitive only to copper oxychloride. While Pandey et al. (2024) found that most isolates of *F. concentricum*, *F. solani*, *F. fujikuroi*, and *F. oxysporum* causing tea dieback in India were insensitive to copper oxychloride. Bachkar et al. (2021) found that *F. incarnatum* causing fruit rot of papaya in India showed sensitivity to carbendazim but insensitivity to mancozeb. In this study, insensitivity to carbendazim and mancozeb was found in *F. compactum*, *F. mianyangense*, and *F. sulawesiense*. In addition, Maniçoba et al. (2023) found that *Fusarium* species (*F. falciforme*, *F. kalimantanense*, *F. pernambucanum*, and *F. sulawesiense*) causing fruit rot of melon in Brazil showed *in vitro* sensitivity to azoxystrobin + fludioxonil and imazalil. Fungicides with a specific mode of action to inhibit fungal growth have been widely used by farmers, as they are known to be more effective in controlling fungal pathogens (FRAC, 2020). Therefore, information on the *in vitro* sensitivity and resistance of fungicides against *Fusarium* species causing fruit rot on muskmelons in this study would be beneficial for *in vivo* applications and for managing this disease in both Thailand and worldwide. Therefore, accurately identifying the fungal agent causing the disease and knowing the fungicide’s sensitivity and resistance will help farmers to prevent damage to melon production. Knowledge of fungicide resistance in fungal pathogens across different countries assists farmers in developing and applying effective control strategies. However, the results from *in vitro* fungicide tests may differ from *in vivo* responses due to environmental conditions and the plant’s metabolism of the fungicide. Therefore, further studies are needed to conduct *in vivo* fungicide sensitivity assays based on the *in vitro* findings. Several previous studies have reported that both the overuse and prolonged application of fungicides contribute to the development of fungicide-resistant strains (Deising et al., 2008; FRAC, 2020; Yin et al., 2023). Reducing fungicide resistance in fungi requires a multifaceted approach that includes using biological control agents, practicing crop rotation, adhering to fungicide application recommendations, and maintaining clean equipment, fields, and storage areas (Lucas, 2017; Corkley et al., 2022; Davies et al., 2021).

Prior to this study, only three *Fusarium* species, *F. equiseti* (Nuangmek et al., 2019), *F. incarnatum* (Wonglom and Sunpapao, 2020), and *F. melonis* (Khuna et al., 2022) have been identified as causing fruit rot in cantaloupes and muskmelons in Thailand. Therefore, this study represents the first report of *F. compactum*, *F. jinanense*, and *F. mianyangense* as novel pathogens causing fruit rot in muskmelons, both in Thailand and worldwide. Additionally, this is the first documented case of *F. sulawesiense* causing postharvest fruit rot on muskmelons in Thailand. Further investigations are required to elucidate the timing of infections caused by fungal pathogens in these fruits. This can be accomplished by tracking the occurrence of disease-causing agents in these fruits throughout various stages of growth in cultivation regions, encompassing both pre- and post-harvest processes, along with the period of preservation after harvest. Additional studies will also be needed to pinpoint the origin of the disease’s inoculum and the meteorological factors influencing infection and disease advancement.

## 5 Conclusions

Fruit rot on muskmelons caused by *Fusarium* is a worldwide disease that frequently occurs throughout fields or during storage. In the current investigation, four pathogenic *Fusarium* species, namely, *F. compactum*, *F. jinanense*, *F. mianyangense*, and *F. sulawesiense*, were isolated from infected muskmelon fruits in northern Thailand. The identification of these fungi involved the analysis of their morphological traits and performing multi-gene phylogenetic analyses. The assessment of pathogenicity for these four fungal species exhibited similar symptoms throughout the artificial inoculation process, as they did during the postharvest storage period. Therefore, the present study is the first in Thailand and worldwide to identify *F. compactum*, *F. jinanense*, and *F. mianyangense* as new causal agents of fruit rot diseases in muskmelons. This is also the first report of postharvest fruit rot on muskmelons caused by *F. sulawesiense* in Thailand. In the fungicide screening test, all fungal isolates showed copper oxychloride sensitivity. However, all isolated shown a insensitivity to metalaxy, carbendazim, mancozeb, and benalaxyl-M + mancozeb. Thus, the findings of this study will improve our understanding of postharvest fruit rot disease of muskmelon and provide insight into developing effective management strategies and prevention to minimize substantial economic losses. Our future study will focus on the epidemiology of postharvest fruit rot disease of muskmelon in different locations of Thailand.

## Data Availability

The datasets presented in this study can be found in online repositories. The names of the repository/repositories and accession number(s) can be found below: https://www.ncbi.nlm.nih.gov/, *cam*: PP758861, PP758862, PP758863, PP758864, PP758865, PP758866, PP758867, PP758868, PP758869; *rpb2*: PP758870, PP758871, PP758872, PP758873, PP758874, PP758875, PP758876, PP758877, PP758878); *tef1-α*: PP758879, PP758880, PP758881, PP758882, PP758883, PP758884, PP758885, PP758886, PP758887.
